# Effect of a parent-supported psychosocial development program on psychosocial outcomes of children aged 1–3 years: a waiting-list randomized controlled trial

**DOI:** 10.1007/s11845-026-04361-8

**Published:** 2026-04-06

**Authors:** Ahu Cirlak, Havva Kacan

**Affiliations:** 1 Department of Nursing, Kulu Faculty of Health Sciences, Selcuk University, Dinek M. Kulu/Konya, Türkiye Turkey; 2 Department of Nursing, Faculty of Health Sciences, Kastamonu University, Kastamonu, Türkiye Turkey

**Keywords:** Nursing, Psychosocial intervention, Randomized controlled trial, Toddlers

## Abstract

**Background:**

Parent-supported psychosocial development programs aim to strengthen the parent-child relationship and promote healthy early childhood development, particularly in settings where access to structured preventive interventions is limited.

Aims To evaluate the effectiveness of a structured parent-supported psychosocial development program on psychosocial outcomes in children aged 1-3 years and to examine its secondary effects on parental anxiety.Methods A waiting-list randomized controlled trial with pretest, posttest, and follow-up assessments was conducted online in Türkiye between November 2024 and February 2025. Forty-three parents of children aged 1-3 years were randomly assigned to the intervention (n=22) or control group (n=21). The six-week structured program consisted of six modules covering 11 topics. Parents in the intervention group received weekly PDF-based educational materials and participated in online group discussions.

**Results:**

Psychosocial development scores at posttest and follow-up were significantly higher in the intervention group compared with the control group (p<0.05), with significant group and group*time interaction effects. No significant differences were found between groups in parental anxiety levels (p>0.05).

**Conclusions:**

The parent-supported program was effective in improving psychosocial outcomes among children aged 1-3 years but did not reduce parental anxiety. Future interventions should consider incorporating strategies specifically addressing parental anxiety to enhance the effectiveness of early psychosocial support programs.

**Trial registration:**

ClinicalTrials.gov Identifier: NCT06951165

**Supplementary Information:**

The online version contains supplementary material available at 10.1007/s11845-026-04361-8.

## Introduction

Early childhood, particularly between the ages of 1 and 3, represents a critical window during which children begin to develop secure attachment, self-regulation, and foundational social skills [1]. According to psychosocial development theory, this stage is marked by a child’s growing efforts toward autonomy. When these efforts are hindered by overprotective, punitive, or restrictive parenting, children may develop feelings of shame and self-doubt [2]. Supporting developmentally appropriate caregiving during this period is essential not only for fostering a healthy personality but also for preventing potential mental health issues later in life [3].

The early years are characterized by rapid developmental changes and high sensitivity to environmental influences. Providing stimulating and nurturing environments alongside informed caregiving can significantly reduce developmental risks. In this context, parent-focused intervention programs play a vital role in systematically enhancing caregivers’ knowledge, skills, and confidence related to childrearing, family relationships, and social responsibilities [3, 4]. Since parents are the primary agents of influence in a child’s life, there is increasing emphasis on strengthening parenting competencies through targeted education [5]. Parenting programs have been shown to improve parental confidence, reduce stress levels, and enhance family dynamics [6, 7]. Global implementation of parenting programs varies, and many have demonstrated effectiveness in improving parenting practices and supporting children’s socio-emotional development [8]. However, a substantial proportion of these interventions have focused on children with behavioral or developmental difficulties [9–11]. Evidence regarding preventive programs targeting parents of typically developing toddlers remains relatively limited.

Structured psychosocial development programs that involve both parents and young children aim to improve children’s social, emotional, and cognitive skills while fostering stronger parent-child relationships [12, 13]. These programs, often delivered through weekly behavioral or relational sessions, offer both informational and experiential learning opportunities to parents and have been linked to gains in children’s emotional regulation, empathy, social communication, and a reduction in problematic behaviors [3].

Despite the increasing recognition of early parent-child interactions in healthy development, intervention studies targeting parents of typically developing children aged 1–3 years remain limited [14–16]. Therefore, evaluating preventive psychosocial development programs implemented with parents of typically developing toddlers may contribute to the limited evidence in this area. Addressing this gap, the present study focuses on parents of typically developing children and evaluates a structured parent-assisted psychosocial development program. Although parental anxiety was not the primary outcome, elevated general anxiety in parents may influence parent-child interactions, contribute to overprotective behaviors, and indirectly affect children’s autonomy development. Early interventions that improve caregiving knowledge and competence may therefore have a potential impact on parental anxiety levels. The primary aim of this study was to examine the effectiveness of the program on children’s psychosocial development. A secondary aim was to explore its potential impact on parental anxiety levels.

## Hypotheses of the study

H1: Children in the intervention group will demonstrate significantly greater improvements in psychosocial development scores over time compared with children in the control group.

H2: Parents in the intervention group will demonstrate lower general anxiety scores, as measured by the Beck Anxiety Scale, over time compared with parents in the control group.

### Methods

#### Design

In this study, a waiting-list randomized controlled trial with a pretest-posttest-follow-up design was conducted with intervention and control groups. The study was conducted between November 2024 and February 2025. The trial was registered at ClinicalTrials.gov during the recruitment phase on 8 January 2025 (NCT06951165).

This study was prepared **i**n accordance with the Consolidated Standards of Reporting Trials (CONSORT 2025) guidelines. (CONSORT 2025) [17].

### Participants

Within the scope of the study, calls were made to parents with children between the ages of 1–3 through social media platforms (Facebook, Instagram, LinkedIn and WhatsApp). In the call, it was stated that a study was conducted for this age group and that there was a “Parent Supported Psychosocial Development Program” offered free of charge. It was stated that the program consisted of 11 different topics for 6 weeks. Parents who wanted to participate in the program applied via the e-mail addresses of the researchers. All parents who requested to participate were asked to complete the “Parent and Child Descriptive Data Form”, “Psychosocial Status Assessment Scale for Parents (1–3 years)” and “Beck Anxiety Scale”. The questionnaires were distributed via Google Forms sent by e-mail. Parents who completed the form within one week were divided into intervention and control groups by randomization method. Inclusion criteria were:

- being a parent whose child was between 1-3 years old,
- using a tablet, computer or smartphone with internet access,
- being fluent in Turkish and volunteering to participate in the study.

Exclusion criteria were:

- the child had a diagnosis such as autism, conduct disorder or hyperactivity,
- the parent was taking psychiatric medication.

### Randomization

Parents who wanted to participate in the study were listed after completing the pre-tests. Randomization was performed by a statistical expert independent of the researcher and the study, using the website www.randomizer.org. Stratified randomization method was used in the study. In order to ensure homogeneity, a total of six strata were created based on parental education level (high school or below; undergraduate or graduate) and number of children (one; two or more) to ensure group homogeneity.

**Fig. 1:**
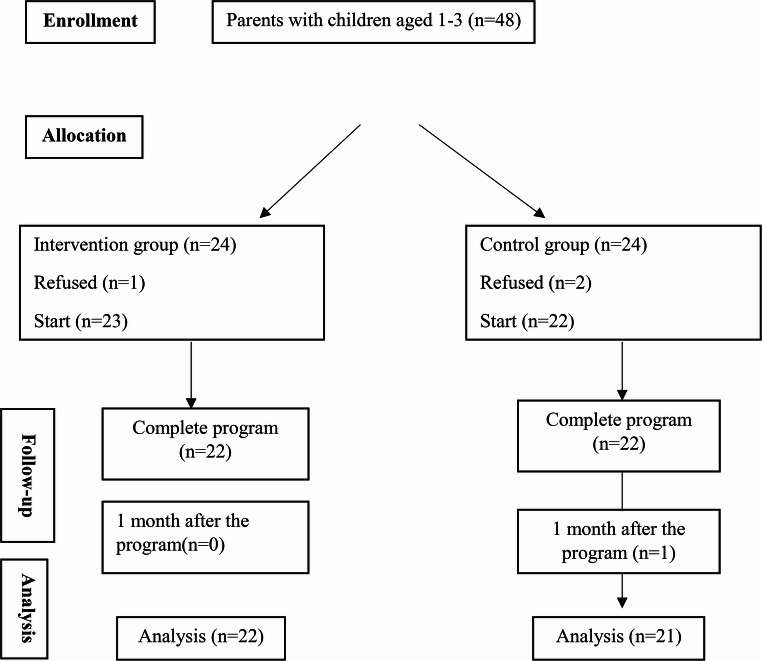
The Consort Flow Chart

Power analysis was used to determine the number of people to be included in the study. In order to determine the sample size to be included in the study, an a priori power analysis was performed using the G*Power 3.1 program. In a similar study [18], the effect size for the change in anxiety level was reported as 0.77. Based on this effect size, the minimum sample size required to detect group*time interactions using an F test (repeated measures ANOVA) with two groups and three measurements (pre-test, post-test, follow-up) was calculated as 20 participants in total (10 per group), assuming a significance level of 0.05 and 95% power (1-β = 0.95). A two-tailed approach was used to account for potential effects in either direction. In the study, it was aimed to reach a total of 48 participants, 24 in each group, by keeping the power of the test high and taking into account possible participant losses. However, three participants left the study two weeks after the beginning of the program and two participants were not included in the study because they did not complete the data collection tools. The study was completed with 43 parents, 22 intervention and 21 control. The consort flow chart of the study is given in Fig. 1.

Blinding: Due to the nature of the behavioral intervention, participant blinding was not feasible. However, allocation concealment was maintained, and statistical analyses were conducted by a blinded statistician. To minimize contamination, intervention and control groups were managed through separate communication channels, and participants had no direct contact with each other during the study period.

### Data collection tools

Parent and Child Descriptive Data Form: The form, which was created by the researchers by reviewing the literature [19–21], consists of 10 questions including mother’s age, mother’s education level, father’s age, father’s education level, child gender, child age, number of children, socioeconomic status, whether the child is diagnosed with autism, conduct disorder, hyperactivity, etc., and whether the parent uses any psychiatric medication.

Psychosocial status assessment scale for parents (1–3 years of age): The scale was developed by Cirlak and Kilicarslan [22] to assess the psychosocial status of children aged 1–3 years by their parents. The scale consists of 25 items. A maximum score of 100 points can be obtained from the scale. A low total score indicates that the child may be at risk for psychosocial problems, while a high score indicates a lower risk for psychosocial problems. When scoring the form, some items are reversed: 3, 6, 8, 9, 10, 11, 12, 13, 14, 15, 19, 20, 21, 24. In this study, the Cronbach Alpha value of the scale was found to be 0.856.

Beck Anxiety Scale: The scale was developed by Beck et al. in [23] and its Turkish validity and reliability was performed by Ulusoy et al. in [24]. The scale aims to measure the severity of anxiety of the individual. Each question is scored between 0 and 3 on a 4-point Likert scale. A total score between 8 and 15 points indicates low level of anxiety, between 16 and 25 points indicates moderate level of anxiety, and between 26 and 63 points indicates high level of anxiety. In this study, the Cronbach’s alpha value of the scale was 0.912.

### Data collection

The study started after ethics committee approval was obtained. WhatsApp groups were created to ensure communication with the intervention and control groups and to convey the day and time information of the program. ‘’Parent Supported Psychosocial Development Program’’ was applied to the intervention group. The control group continued their routine childcare. At the end of the study, the same program was applied to them. Data were collected at three different times for the intervention and control groups: before the program (T0), immediately after the program (T1) and one month after the program (T2).

## Intervention

### Parent supported psychosocial development program

The content of the program was prepared in line with the current literature to meet the needs of parents with children aged 1–3 years to support the psychosocial development of their children, to acquire knowledge and skills. The training modules prepared by the researchers were submitted to expert opinion and the final content of the program was determined in line with the feedback received. Expert opinion was obtained from a pediatrician and six academicians in the field of pediatrics and mental health nursing. The program is planned to consist of a total of six modules. Care was taken to ensure that the modules were illustrated with clear information. It was explained to the parents that they should participate regularly in the program and take an active part in at least four modules. The prepared modules were sent to the parents via e-mail in PDF format at the beginning of the week. Participation in the program was monitored through the online discussion sessions, which required parents to summarize and reflect on the content of the PDF modules they read. Active participation in these sessions was considered a proxy for engagement with the intervention materials. Parents were required to participate in at least four of the six modules, a threshold determined through expert consensus to ensure sufficient exposure to the program content while accommodating scheduling constraints. Although individual module completion data were not formally recorded for all participants, all parents met the minimum participation requirement, and no systematic differences in outcomes were observed related to variation in participation. At the end of the week, the parents who read the modules discussed the modules they read with the researchers in groups of at least five and at most eight people by choosing one of the two different hours offered to them online. The online sessions were planned to last a maximum of 60 min. During the training, parents were encouraged to interact with each other by discussing how they could realize the applications for their children through the PDFs they read. Parents were first asked about their experiences within the scope of the weekly topic determined by the researchers. Afterwards, correct behaviors were reinforced and feedback was given for incorrect ones. In addition, parents were informed that they could receive counseling from the researchers on educational topics whenever they wanted.

### Training titles

1.What is Psychosocial Development? Introduction of psychosocial development of children in this age group. 2.Social Communication and Language Development: Supporting children’s social skills and language development. 3.Emotional Expression and Management of Emotions: Gaining the skills of recognizing, expressing and managing emotions. 4.Empathy and Social Skills: Ways of understanding, sharing and empathizing with the feelings of others. 5.The Role of Play and Social Interaction: Learning through play and the development of social relationships. 6.Behavior Management: Positive Discipline Methods: Setting limits, patience and positive discipline methods. 7.Family Communication and Support: Cooperation between parents, family role models and healthy communication with the child. 8.Self-Esteem and Self-Confidence Development: Ways to strengthen the child’s self-esteem and self-confidence. 9.Crisis Moments and Coping Skills: Methods of coping with difficult emotions such as anger and frustration. 10.Technology and Screen Time Management: Screen time and the effects of the digital world for children aged 1–3 years. 11.Meeting the Parent’s Own Psychosocial Needs: Being aware of your own stress management and emotional needs as a parent.

Modules 7–11 of the program specifically target parents’ own psychosocial needs, such as family communication, self-confidence, coping with difficult emotions, and managing stress. By enhancing parental knowledge, self-efficacy, and emotional awareness, these components are expected to indirectly influence parental anxiety, supporting the study’s secondary aim. The program does not include clinical interventions for anxiety, but improving parents’ coping and problem-solving skills provides a theoretical pathway for reducing anxiety levels.

### Ethical considerations

The study was approved by the Non-Interventional Clinical Research Ethics Committee of XXX University (10.09.2024, 2024-KAEK-71). Permission was obtained from the authors who conducted the validity and reliability study for the scales used in the study. After the participants read the informed consent form at the beginning of the study, they continued by checking “yes” in the statement “I agree to be informed about the research and to participate in the study.” Those who approved the informed consent form were included in the study. The study was conducted in accordance with the Declaration of Helsinki.

### Control group

Parents in the control group continued with their traditional childcare. Data were collected at the same time (T0, T1 and T2) as the intervention group. Data collection tools were created through Google Forms and sent by email. After the study, the PDFs were sent to the parents in the control group and once a week, at a time determined by them, discussions were held online to put the information in the modules into practice.

### Statistical analysis

The data obtained in the study were analyzed using SPSS (Statistical Package for Social Sciences) for Windows 22.0 program. Number, percentage, mean, standard deviation were used as descriptive statistical methods in the evaluation of the data. Differences between the rates of categorical variables in independent groups were analyzed with Chi-Square tests. Kurtosis and Skewness values were analyzed to determine whether the research variables were normally distributed. In psychosocial status variables, skewness values ranged between − 0.89 and − 0.49; kurtosis values ranged between − 0.65 and 0.67. In anxiety variables, skewness values ranged between − 0.39 and 1.33; kurtosis values ranged between 0.85 and 1.45. Independent groups t-test was used to compare quantitative continuous data between two independent groups. Repeated-measures ANOVA was used to compare measurements over time.

## Results

Table 1 shows the descriptive characteristics of the participants in the intervention and control groups. Maternal education level, paternal education level, income status, number of children, child gender, parental and child age were found to be similar. There was no statistically significant difference between the groups (*p* > 0.05).

**Table 1:** Distribution of Parents According to Their Descriptive Characteristics

		Intervention group		Control group		*p*
		*n*	%	*n*	%	
Mother’s education	High school	4	18.2	3	14.3	X^2^=5.373 *p* = 0.146^a^
	Associate degree	4	18.2	4	19.0	
	Bachelor’s degree	11	50.0	5	23.8	
	Graduate degree	3	13.6	9	42.9	
Father’s education	High school	5	22.7	6	28.6	X^2^=1.211 *p* = 0.750^a^
	Associate degree	2	9.1	3	14.3	
	Bachelor’s degree	12	54.5	8	38.1	
	Graduate degree	3	13.6	4	19.0	
Income level	Income less expenses	3	13.6	4	19.0	X^2^=2.435 *p* = 0.296^a^
	Income equals expenses	10	45.5	13	61.9	
	Income exceeds expenses	9	40.9	4	19.0	
Number of children	Only	15	68.2	14	66.7	X^2^=0.011 *p* = 0.586^b^
	Two or more children	7	31.8	7	33.3	
Gender of child	Boy	10	45.5	9	42.9	X^2^=0.029 *p* = 0.554^b^
	Girl	12	54.5	12	57.1	
		***x̄***	***SD***	***x̄***	***SD***	***t*** **/** ***p*** ^**c**^
Mother’s age		29.910	3.421	30.670	3.679	-0.700/0.488
Father’s age		31.910	3.517	33.140	3.692	-1.122/0.268
Child’s age (month)		21.320	7.606	21.050	6.837	0.122/0.903

^a^Chi-square Analysis; ^b^Fisher Exact; Independent Groups t-test

Table 2 shows the comparison of psychosocial status scale scores according to the groups. There was no statistically significant difference between the pre-test scores of the intervention and control groups (*t* = 0.796; *p* = 0.431). However, when the post-test scores were compared, it was seen that the scores of the intervention group were statistically significantly higher than the control group (*t* = 4.066; *p* < 0.001). Similarly, a statistically significant difference was found between the groups in the follow-up test (*t* = 2.605; *p* = 0.013). According to repeated measures ANOVA results, the time effect was not statistically significant (*F* = 1.855; *p* = 0.168; *η²*=0.043, small), but the group effect was statistically significant (*F* = 8.250; *p* = 0.006; *η²*=0.168, medium). In addition, the group*time interaction is statistically significant (*F* = 4.032; *p* = 0.027; *η²*=0.090, medium).

**Table 2:** Comparing Psychosocial Status Measurements Between the Intervention and Control Groups

	Intervention group (*n* = 22)		Control group (*n* = 21)		t^a^	*p*
	x̄	SD	x̄	SD		
Psychosocial Status (T0)	79.091	9.329	76.762	9.869	0.796	0.431
Psychosocial Status (T1)	85.273	4.832	75.571	10.043	4.066	0.001
Psychosocial Status (T2)	82.364	8.127	75.095	10.104	2.605	0.013
Time	*F* = 1.855; *p* = 0.168; *η²*=0.043					
Group	*F* = 8.250; *p* = 0.006; *η²*=0.168					
Group*Time	*F* = 4.032; *p* = 0.027; *η²*=0.090					

^**a**^Independent Groups t-test; ^**b**^Repeated Measures Anova Test

Beck anxiety scale scores were compared according to the groups in Table 3. There was no statistically significant difference between the pretest, posttest and follow-up test scores of the intervention and control groups (*p* > 0.05). In the pretest, the mean anxiety score of the intervention group was 11.227, while that of the control group was 9.000 (*t* = 0.932; *p* = 0.357). Similarly, no statistically significant difference was found between the groups in the post-test and follow-up test (*p* = 0.367 and *p* = 0.777, respectively). According to repeated measures ANOVA results, time effect (*F* = 0.161; *p* = 0.792; *η²*=0.004, negligible), group effect (*F* = 0.256; *p* = 0.615; *η²=*0.006, negligible) and group*time interaction (*F* = 1.268; *p* = 0.281; *η²*=0.030, small) were not statistically significant.

**Table 3:** Comparing Beck Anxiety Measurements Between the Intervention and Control Groups

	Intervention group (*n* = 22)		Control group (*n* = 21)		t^a^	*p*
	x̄	SD	x̄	SD		
Anxiety (T0)	11.227	6.879	9.000	8.729	0.932	0.357
Anxiety (T1)	10.546	8.122	8.429	7.040	0.911	0.367
Anxiety (T2)	9.364	10.957	10.238	9.027	− 0.285	0.777
Time	*F* = 0.161; *p* = 0.792; *η²*=0.004					
Group	*F* = 0.256; *p* = 0.615; *η²*=0.006					
Group*Time	*F* = 1.268; *p* = 0.281; *η²*=0.030					

^**a**^Independent Groups t-test; ^**b**^Repeated Measures Anova Test

### Harms

Harms were defined as any emotional discomfort, increased stress or anxiety, or feelings of inadequacy related to parenting that could arise from participating in the online educational sessions. Harms were assessed systematically using a brief self-report form administered to parents after each session, which inquired about any emotional burden, stress, or discomfort experienced. No serious adverse events or harms were reported during or after the intervention.

## Discussion

Psychosocial development forms the basis of the individual’s lifelong social relationships, emotional balance and personality structuring. The healthy course of this development depends on the foundations laid in the first years of life [16]. 1–3 years of age is a period in which critical skills such as adapting to the environment, establishing secure attachment and expressing emotions are shaped. In this process, parents’ guidance and support play a decisive role in the child’s psychosocial development [25]. This study presented a structured intervention program for parents to support children’s psychosocial development and assessed both children’s developmental level and parents’ anxiety level. The findings revealed that children’s psychosocial development levels increased significantly after the intervention. In the scales completed by the parents in the intervention group, children’s scores in the post-test and follow-up tests were statistically significantly higher compared to the control group. In addition, the group and time interaction was significant, indicating that the intervention had a lasting and strengthening effect. These results are consistent with similar interventions in the literature. The Effective Parenting Program developed by Pala et al. [26] also led to positive improvements in the attitudes of parents with children aged 2–6 years and in the emotional regulation skills of children [26]. Panina and Mamarina’s (2023) “Baby” program offers structured pedagogical and psychological support for 1–3 year old children and has positive effects on children’s social motivation, self-regulation and cognitive skills [1]. These findings support the contributions of the intervention to child development in the current study. Similarly, the Family Foundations program was found to have positive effects on emotional adjustment in children aged 1–3 years and reduced stress levels in parents. Psychosocial development develops rapidly during early childhood. Therefore, this period has been recognized as a critical period for the development of various mental pathologies later in life. Early assessment and intervention can reduce parental anxiety and make parents more confident and successful in problem-solving skills during child-rearing practices. Even simple assessment procedures can have positive contributions to child-parent relationships [15, 20].

In our study, no significant difference was found in terms of parents’ anxiety levels. This may be due to the fact that the main focus of the intervention program was the developmental needs of children and did not include a specific content for parental anxiety. In addition, the limited duration may not be sufficient to have the expected effect on the mental state. Another possible explanation for the absence of a significant effect on parental anxiety may be the relatively low baseline anxiety levels observed in both groups. When initial scores are already low, the potential for further reduction may be limited, which can lead to a possible floor effect. Future studies including parents with higher baseline anxiety levels may provide a clearer understanding of the potential impact of such interventions on parental anxiety. Some studies in the literature have observed more significant changes in stress and anxiety levels. The SOLACE program conducted by Lodder et al. [27] is an intervention developed for parents of children with autism and provided positive changes in parents’ mental health, self-esteem and self-compassion levels [27]. The cognitive-behavioral-based parent-focused intervention developed by Cartwright-Hatton et al. [28] not only reduced anxiety symptoms in children, but also had positive effects on parents’ own anxiety [28]. Similarly, the online modular intervention program conducted by Dunn et al. [29] provided significant improvements in the mental health of both parents with high anxiety levels and their children [29]. In this context, the lack of effect on parental anxiety in the current study can be explained by the fact that the intervention content was not specific to stress management. Nevertheless, the fact that parents came together online and shared their experiences created a potential psychological support mechanism in terms of peer support. However, these effects may not have been immediately measurable in short-term applications. As Feinberg et al. (2010) emphasized, interventions that improve parenting skills and directly target mental health can create more lasting change [30].

In conclusion, this study showed that a structured program developed for parents to support the psychosocial development of children aged 1–3 years had positive effects on children’s developmental levels. However, no significant change was found in parents’ anxiety levels, which points to the importance of content directly targeting parental mental health in future studies.

### Strengths and limitations

One of the key strengths of this study is the online delivery of the program, which enabled the participation of parents from various regions of Türkiye with diverse cultural backgrounds. This geographical diversity enriched peer learning and interaction among participants. Additionally, providing the modules in PDF format allowed parents to revisit the content at their own pace, which may have supported learning retention.

A limitation of this study is that participants were recruited voluntarily via social media platforms (Facebook, Instagram, LinkedIn, and WhatsApp), which may introduce self-selection bias. Parents who responded are likely to be more interested in child development, highly motivated, and digitally literate than the general population, which may affect the generalizability of the findings.

Despite these strengths, several limitations should be considered. First, the relatively small sample size limits the generalizability of the findings. Second, although online group sessions were a core component of the intervention, some parents chose to follow the program only through the written materials without actively participating in the sessions, which may have reduced the overall intensity of the intervention. One limitation of the psychosocial development measure is that it was developed by one of the study authors, which may introduce potential measurement bias, although its validity and reliability have been previously established.

Another limitation is that the follow-up assessment was conducted one month after the intervention. While this provides insight into short-term sustainability, it may not be sufficient to evaluate lasting developmental effects in early childhood. Future studies with longer follow-up periods are recommended to assess the durability of the intervention effects.

## Conclusion

In this study, the results indicate that the parent-assisted intervention had a positive effect on the psychosocial development of children aged 1–3 years in the intervention group, but did not have a significant impact on parents’ anxiety levels. Future research is recommended to include longer-term programs specifically designed to address parental anxiety, incorporating cognitive-behavioral techniques. The findings of this study suggest that parent-assisted psychosocial development programs can be used as a sustainable and effective early intervention strategy. Additionally, such programs can be adapted into individual or group activities for children with special needs and their families.

## Supplementary Information

Below is the link to the electronic supplementary material.

Supplementary Material 1.

Supplementary Material 2.
